# Proposal of a novel pipeline involving precise bronchoscopy of distal peripheral pulmonary lesions for genetic testing

**DOI:** 10.1038/s41598-022-24372-6

**Published:** 2022-11-17

**Authors:** So Takata, Kotaro Miyake, Daichi Maeda, Kazue Hatake, Izumi Nagatomo, Takayuki Shiroyama, Kentaro Masuhiro, Moto Yaga, Yuya Shirai, Yuichi Mitsui, Shinichi Yachida, Atsushi Kumanogoh

**Affiliations:** 1 Department of Respiratory Medicine and Clinical Immunology, Graduate School of Medicine, Osaka University, 2-2 Yamadaoka, Suita, Osaka 565-0871 Japan; 2 Department of Cancer Genome Informatics, Graduate School of Medicine, Osaka University, Suita, Osaka Japan; 3 Department of Molecular and Cellular Pathology, Kanazawa University, Kanazawa, Japan; 4 Department of Center for Cancer Genomics and Personalized Medicine, Graduate School of Medicine, Osaka University, Suita, Osaka Japan; 5 Department of Immunopathology, World Premier International Research Center Initiative, Immunology Frontier Research Center, Osaka University, Suita, Osaka Japan; 6 Integrated Frontier Research for Medical Science Division, Institute for Open and Transdisciplinary Research Initiatives, Osaka University, Suita, Osaka Japan; 7 Center for Infectious Diseases for Education and Research, Osaka University, Suita, Osaka Japan; 8 Japan Agency for Medical Research and Development—Core Research for Evolutional Science and Technology, Osaka University, Suita, Osaka Japan; 9 Center for Advanced Modalities and DDS, Osaka University, Suita, Osaka Japan

**Keywords:** Cancer genomics, Cancer imaging

## Abstract

Next-generation sequencing (NGS) has become increasingly more important for lung cancer management. We now expect biopsies to be sensitive, safe, and yielding sufficient samples for NGS. In this study, we propose ultraselective biopsy (USB) with sample volume adjustment (SVA) as a novel method that integrates an ultrathin bronchoscope, radial probe endobronchial ultrasound, and the direct oblique method for ultraselective navigation, and adjustment of sample volume for NGS. Our purpose was to estimate the diagnostic potential and the applicability of USB-SVA for amplicon-based NGS analysis. The diagnostic yield of bronchoscopy in forty-nine patients with malignant peripheral pulmonary lesions (PPLs) was retrospectively analyzed, and amplicon-based NGS analysis was performed on samples from some patients using USB. The diagnostic yields of distal PPLs in the USB group were significantly higher than those in the non-USB group (90.5% vs. 50%, respectively, *p* = 0.015). The extracted amounts of nucleic acids were at least five times the minimum requirement and the sequence quality met the criteria for the Oncomine™ Target Test. Only the tumor cell content of some samples was insufficient. The feasibility of the pipeline for USB, SVA, and amplicon-based NGS in distal PPLs was demonstrated.

## Introduction

Next-generation sequencing (NGS)-based molecular profiling plays a critical role in the management of lung cancer. Therefore, it is crucial to establish a pipeline from lung cancer biopsy to NGS analysis, which has a high success rate and uses minimally invasive techniques. Bronchoscopy is a safe method for biopsy of peripheral pulmonary lesions (PPLs). The utility of cryobiopsy for PPLs has been reported in obtaining a larger sample for genetic testing^1^. However, distal PPLs are not recommended for cryobiopsy because of the risk of pneumothorax^2^. Therefore, a safe method is needed to obtain distal PPL samples with safety and yield sufficient for genetic testing.

Recently, we established the direct oblique method (DOM) as an approach for ultraprecise bronchoscopic planning^3,4^. DOM is a manual technique used in computed tomography (CT) analysis of general-purpose CT scans, but it is fast and more precise than the automatic analysis of conventional virtual bronchoscopic navigation (VBN) systems^3^. The main scheme of DOM is based on the use of virtual bronchoscope (VB)-like oblique CT images instead of a VB, which can use the entire bronchi detected by CT as its routes of entry. We demonstrated that the median number of bronchial generations prior to each terminal tip with DOM was ten, whereas that of VBN was five^4^. To increase its user-friendliness, DOM was integrated into the Zionstation2 software (Ziosoft, Tokyo, Japan)^3^. Given that DOM is an effective method for navigating the periphery of the lung, ultrathin bronchoscopy and DOM are theoretically a good combination.

This is a proof-of-principle study of a new approach for biopsy of small PPLs. We propose ultraselective biopsy (USB) as a method that integrates an ultrathin bronchoscope, radial probe endobronchial ultrasound (r-EBUS), and DOM for ultraselective navigation. Additionally, for efficient use of even the smaller specimens, we conducted sample volume adjustment (SVA), which uses more sections if the sample size is small. The purpose of this study was to estimate the diagnostic yield of methods that include USB and evaluate the applicability of USB-SVA for amplicon-based NGS analysis using the Oncomine^™^ Target Test (OTT; Thermo Fisher Scientific, Carlsbad, CA, USA), which is a cancer gene panel test of 46 cancer-associated genes (Fig. S1).

## Methods

### Study design

First, we estimated the diagnostic yield of methods that included USB. Second, we prospectively conducted an OTT analysis using samples obtained by the USB-SVA. This study complies with the principles of the Declaration of Helsinki. This study was approved by the Research Ethics Committee of Osaka University (protocol #864). Written informed consent for study participation was obtained from all the patients.

### Bronchoscopic examination

**Figure 1:**
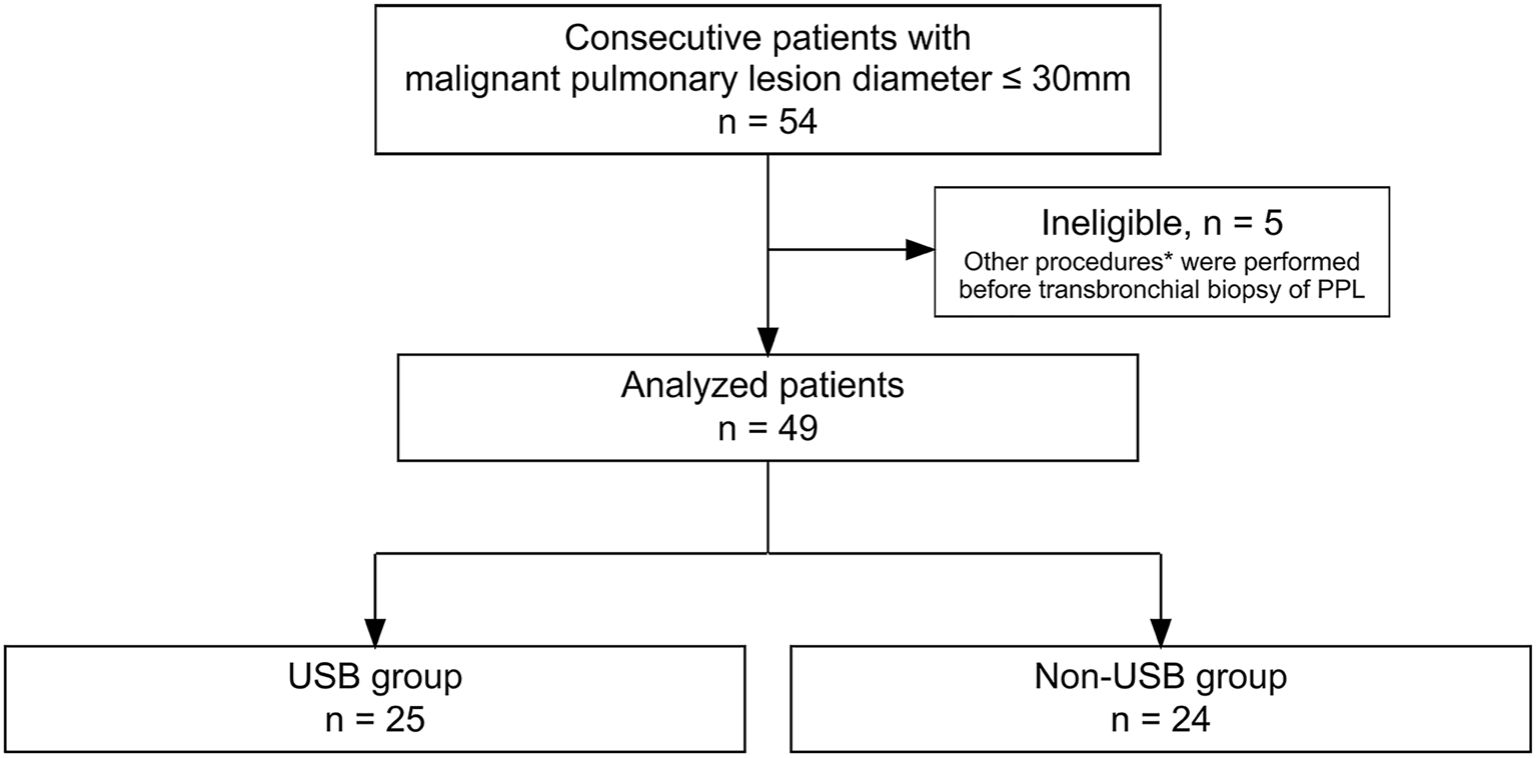
Flow diagram. Forty-nine patients who underwent bronchoscopy and had a malignant pulmonary peripheral lesion (PPL) with a diameter of ≤ 30 mm were retrospectively analyzed for diagnostic yield. *Other procedures consisted of endobronchial ultrasound–guided transbronchial needle aspiration and collection of bronchial alveolar lavage fluid.

Seventy-two consecutive patients who underwent bronchoscopy between August 2018 and March 2019 at Osaka University Hospital were retrospectively enrolled. Each patient had PPL with a diameter of 30 mm or less. As the purpose of this study was evaluating specimen procurement for precision medicine, we focused on patients with malignant lesions. Patients with benign (n = 13) or undiagnosed lesions (n = 5) were excluded. Of the 54 patients, five cases in which other procedures had already been performed were excluded because of potential contamination, and 49 patient cases were analyzed (Fig. 1). The diagnostic yield was compared between methods that included USB (USB group), in which diagnosis was made using a combination of an ultrathin bronchoscope, r-EBUS, and DOM, and methods that did not include USB (non-USB group). Then, the diagnostic yield was stratified by the fifth bronchial generation prior to the target lesion because the median number of bronchial generations reached by thin bronchoscopy was reported to be four^5–7^. Both histological and malignant cytological findings were considered when determining whether bronchoscopy results were positive.

The bronchoscopic method used was as follows. An ultrathin bronchoscope (distal end diameter, 3.0 mm; working channel [WC] diameter, 1.7 mm; MP290; Olympus, Tokyo, Japan), a thin bronchoscope (distal end diameter, 4.1 mm; WC diameter, 2.0 mm; P290F; Olympus), and a standard-sized bronchoscope (distal end diameter, 5.9 mm; WC diameter, 3.0 mm; 1 T-Q290; Olympus) were used. r-EBUS was performed using an endoscopic ultrasonography system (EU-ME2 PREMIER PLUS; Olympus) equipped with two mechanical radial-type probes: a 20 MHz probe (XUM-S2017R; Olympus) and a 1.4 mm ultrasonic probe (UM-S20–17S; Olympus). For transbronchial biopsy, small-sized forceps (diameter, 1.5 mm; open-width diameter, 4.3 mm; FB-433D; Olympus) were chosen when an ultrathin bronchoscope or a thin bronchoscope with EBUS-GS using a 1.95 mm GS (SG-200C; Olympus) was used. Standard-sized forceps (diameter, 1.9 mm; open-width diameter, 5.0 mm; FBB211-D; Olympus) were used with a standard-sized bronchoscope or a thin bronchoscope without EBUS-GS. Biopsies with standard-sized forceps were performed after biopsies with small forceps as appropriate. Brushing was routinely performed after the transbronchial biopsy. In addition, a curette was used as a guiding device when the r-EBUS device could not reach the target lesion. Moreover, transbronchial needle aspiration was performed with a thin-or standard-sized bronchoscope when the r-EBUS device was not within range of the target. Manual bronchoscopic planner was performed using one of the following: DOM on Zionsation2, DOM on SYNAPSE VINCENT, and virtual bronchoscopic planner was performed by VINCENT-BFsim. If appropriate, the bronchial pathway was projected onto the X-ray-like image reconstructed from the CT data. For sedation, the larynx was sprayed 10 times with an 8% lidocaine spray, and midazolam and fentanyl were administered intravenously. If there was a risk of desaturation or bleeding, or if the use of multiple bronchoscopes was planned, the patient was intubated.

### The DOM

The DOM was conducted as previously reported^3^. Briefly, the route to the target was manually written into the CT images by the operators. Fine bifurcations were carefully evaluated with longitudinal and transverse oblique CT images of the bifurcation. In the periphery, VB-like oblique CT images were used instead of VB (see Supplementary Video). In this study, DOM was conducted using a DOM-integrated Ziostation2 CT bronchoscopic navigation system. SYNAPSE VINCENT, a general-application CT viewer, was also used for DOM.

### Measurement of sample area and the OTT

Assuming that small samples were obtained using only small forceps, we prospectively assessed whether the OTT could be successfully performed using the appropriate amount of nucleic acid extracted from the samples obtained by USB-SVA. The idea of SVA is to ensure nucleic acid extraction amount by increasing the number of slides based on the sample size calculation. Formalin-fixed paraffin-embedded (FFPE) tissue samples from lung biopsies were subjected to OTT.

An additional hematoxylin and eosin section was prepared in each case for use by a surgical pathologist (D.M.) to evaluate the tumor cell content in each section and to measure tissue areas. Although OTT is generally performed only when the tumor cell content is 20% or higher, we did not exclude samples with lower values because the aim of this study was to assess whether sequencing could be appropriately performed even with small samples obtained by USB-SVA, regardless of the tumor cell content. We measured the tissue areas using the CellSens Standard image analysis software (Olympus) (Fig. S2). When multiple biopsy samples were obtained, the areas of all the samples were added to obtain the total sample area. Based on this area, we determined the number of 10 μm-thick sections that would undergo DNA/RNA extraction and subsequent sequencing, with a target cumulative area of at least 25 mm^2^ and ideally 50 mm^2^. The targeted number of sections was doubled in a few exceptional cases, in which tissue cellularity was extremely low. We then used whole sectioned samples that were not placed on glass slides and were not obtained by removing tissue by scraping or macro-dissection. Thereafter, the sample processing and sequencing procedures were identical to those specified in the manufacturer’s instructions for the Oncomine^*™*^ Dx Target Test, which has already been used in clinical practice. The amounts of extracted DNA and RNA were evaluated to determine if they were above the threshold (Table S1) for appropriate sequencing (10 ng for both DNA and RNA).

### Statistical methods

To analyze the diagnostic yield, Fisher’s exact test or the chi-square test was used for categorical factors, whereas Student’s t-test and the Mann–Whitney U test were used for continuous factors, as appropriate. Multivariate logistic regression analyses were performed to determine the association between these factors and diagnostic yield.

All statistical analyses were performed using the R ver. 3.6.1 software (http://www.R-project.org; R Foundation for Statistical Computing, Vienna, Austria). Statistical significance was set at *P* < 0.05.

## Results

### Diagnostic yield of USB

The patient characteristics are shown in Table 1. There were no significant differences between the two groups, except that the target tended to be smaller and more peripherally located in the USB group. The details of bronchoscopy-related factors of USB and non-USB groups are shown in Table S2. Regarding complications, one patient in the non-USB group developed a minor pneumothorax, whereas no complications were observed in the USB group.

**Table 1:** Patient characteristics.

	USB group (N = 25)	Non-USB group (N = 24)	*P* value
Age, years (mean ± SD)	69.84 ± 8.92	72.21 ± 10.43	0.397
Male (%)	12 (48.0)	15 (62.5)	0.464
Diameter of lesion, mm (mean ± SD)	16.36 ± 6.90	20.33 ± 6.38	0.042
**Appearance on CT (%)**			0.199
Ground glass opacity	3 (12.0)	1 (4.2)	
Part solid	2 (8.0)	6 (25.0)	
Solid	20 (80.0)	17 (70.8)	
†Bronchial generation (median [IQR])	7.00 [6.00–8.00]	6.00 [4.00–7.00]	0.048
**Final diagnosis (%)**			0.331
Adenocarcinoma	17 (68.0)	16 (66.7)	
Large cell lung carcinoma	0 (0.0)	1 (4.2)	
Malignant lymphoma	0 (0.0)	1 (4.2)	
Metastatic lung carcinoma	3 (12.0)	0 (0.0)	
Non-small cell lung carcinoma, not otherwise specified	0 (0.0)	1 (4.2)	
Small cell lung carcinoma	0 (0.0)	1 (4.2)	
Squamous cell carcinoma	4 (16.0)	4 (16.7)	
Undifferentiated carcinoma (unknown origin)	1 (4.0)	0 (0.0)	

*CT* computed tomography; *DOM* direct oblique method; *IQR* interquartile range; *SD* standard deviation.

^†^Bronchial generation nearest or leading to the target, counted by DOM. Fisher’s exact test or the chi-square test was performed for categorical variables, as appropriate, and Student’s t-test and the Mann–Whitney *U* test were performed for continuous variables, as appropriate.

The overall diagnostic yield was better in the USB group than in the non-USB group, but the difference was not significant (88% vs. 71%, respectively; *p* = 0.171; Table 2). We focused on targets located peripherally to the fifth bronchi (hereafter referred to as “distal PPLs”) because thin bronchoscopy has been reported to reach a median of four bronchial generations^5–7^. For these distal PPLs, the diagnostic yield was significantly higher in the USB group than in the non-USB group (91% vs. 50%, *p* = 0.015) (Table 2). The background characteristics of the subgroups are shown in Table S3. The procedure time was longer in the USB group than that in the non-USB group. The result of logistic analysis showed that the USB group had a significantly higher diagnostic yield (odds ratio, 9.92; 95% confidence interval, 1.68–94.98; *p*-value, 0.02); procedure time was not significantly related to the higher yield result (Table S4). Additionally, samples were separately obtained by small-sized and standard-sized forceps in six patients from the USB group, three patients were diagnosed only by small forceps with an ultrathin bronchoscope, while one patient was diagnosed only by standard-sized forceps with a thin bronchoscope (Table S5). These results imply that small distal PPLs are sometimes obtained using only small-sized forceps.

**Table 2:** Diagnostic yield.

		USB group	Non-USB group	*P* value
Total		88% (22/25)	71% (17/24)	0.171
Lesion diameter	†Bronchial generation			
≤ 30 mm	≤ 5	75% (3/4)	100% (10/10)	0.286
	> 5	91% (19/21)	50% (7/14)	0.015*
≤ 20 mm	≤ 5	66% (2/3)	100% (4/4)	0.429
	> 5	88% (15/17)	38% (3/8)	0.017*

Values shown are percentages (diagnosed numbers/total number). USB, ultraselective biopsy.

*P-value < 0.05.

^†^Bronchial generation nearest to or leading to the target, counted by direct oblique method (DOM).

### OTT analysis of USB samples

Based on the diagnostic yield of USB, we focused on patients with distal small PPLs for whom USB was particularly effective; this means that, since small-sized forceps are used, only small samples could be obtained. From the 19 diagnosed patients with distal small PPLs in the USB group, ineligible patients were excluded: samples not separated using different-sized forceps (n = 4), diagnosis only by cytology (n = 3), diagnosis only by standard-sized forceps (n = 1), and samples expected to be required for clinical practice (n = 1). The remaining 10 patients, whose samples were obtained using USB with ultrathin bronchoscopy, were analyzed.

**Figure 2:**
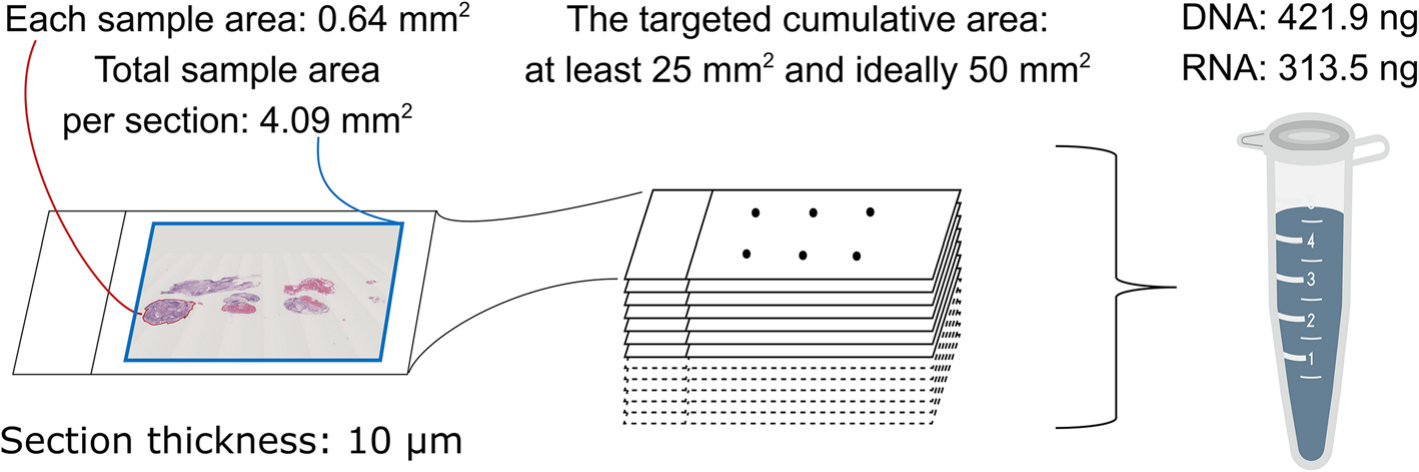
The results of nucleic acid extraction. The median sample area, total sample area, and median quantities of DNA and RNA are presented as representative data. As the targeted area was at least 25 mm^2^ and ideally doubled, the calculated number of sections in this figure was at least seven and, ideally, 13. The quantity of extracted nucleic acid was greater than required (10 ng, according to the manufacturer’s instructions).

The median area of individual samples was 0.64 mm^2^ (range, 0.03–2.42 mm^2^; Table S3). The median number of biopsied samples was 6.5 (range, 4–9; Table S6), and the median number of sections was 12 (range, 10–25; Fig. 2). The cumulative area of each sample reached the target value (median: 49.1 mm^2^; range: 23.9–76.7 mm^2^; Table S6). A sufficient amount of both DNA (median: 421.9 ng, range: 102.1–802.9) and RNA (median: 313.5 ng, range: 57.9–1274.7) were extracted, and the sequence quality for both DNA and RNA met the criteria for OTT (Table S6). We successfully obtained NGS results without a shortage of nucleic acids in any sample, although the number of samples that succeeded in OTT analysis was six because the tumor cell content in four patients was less than 20%. One or more genetic aberrations were detected in five cases (Table 3).

**Table 3:** Results of OTT analysis.

No	Mutation detected by OTT	Histologic finding	Tumor cell content (%)	Sum of specimen area (mm2)	Sectioned count	Cumurative area (mm^2^)	DNA total (ng)	RNA total (ng)	Sequence QC
1	PIK3CA mutation	Squamous cell carcinoma	50	5.28	12	63.4	667.42	481.44	Passed
2	ERBB3 mutation	Squamous cell carcinoma	30	7.67	10	76.7	616.42	1274.7	Passed
3	EGFR mutation (p.E746_A750del) PIK3CA mutation	Adenocarcinoma	20	5.01	12	60.1	456.76	469.68	Passed
4	no mutation*	Adenocarcinoma	10	5.8	20†	116	371.62	323	Passed
5	KRAS mutation (p.G12C)*	Adenocarcinoma	5	2.44	20†	48.8	164.65	192.28	Passed
6	no mutation*	Adenocarcinoma	3	1.62	25	40.5	763.84	514.56	Passed
7	no mutation*	Adenocarcinoma	1	3.27	10	32.7	102.08	57.92	Passed
8	EGFR mutation (p.E746_A750del) ERBB2 amplification	Adenocarcinoma	20	4.5	12	54	387	271.66	Passed
9	BRAF mutation (G469A)	Adenocarcinoma	20	3.69	12	44.3	299.2	303.96	Passed
10	no mutation	Malignant tumor	30	1.33	18	23.9	802.9	67.65	Passed

*OTT* Oncomine™ Target Test.

*Tumor cell content in these patients was lower than the predetermined threshold.

^†^In samples from these patients, cellularity was poor; thus, the section count doubled.

## Discussion

This report demonstrated that the pipeline from ultraselective bronchoscopy to cancer panel testing is useful for small distal PPLs. This pipeline utilized OTT, an amplicon-based NGS test in which the number of sections is based on the sample size. Although the number of samples obtained by USB was small, the amount of extracted nucleic acids exceeded the OTT quantity criteria in all patients. Genetic mutation testing is becoming increasingly important for lung cancer management, and the usefulness of r-RBUS with guide sheath, conventional virtual bronchoscopic planners (e.g., LungPoint, etc.), and electromagnetic navigation systems has been reported^8–10^. Our results indicate that ultraselective bronchoscopy using ultrathin bronchoscopy combined with precise bronchoscopic planner (DOM) and r-EBUS is also useful for obtaining the volume of small distal PPL samples sufficient for NGS-based tumor analysis, displaying a fairly successful instance of precision medicine by adjustment, from a pathological point of view.

Surprisingly, we were able to extract adequate amounts of nucleic acids for OTT from small samples obtained using USB. While only 10 ng of both DNA and RNA was required according to the manufacturer’s instructions, we were able to extract at least five times the amount. This may have resulted from the strict measurement of tissue areas and the adjustment of the number of sections to achieve an appropriate sample volume. Sakaguchi et al. showed that small specimen obtained by small forceps could be the factor which lessens the success rate of OTT^11^. However, our results imply that, regarding sequencing failure due to quantity in clinical practice, the size of samples obtained by small-sized forceps is not a problem; rather, the amount of input to nucleic acid extraction is the problem.

Our study demonstrated that for small distal PPLs with diameters less than 30 mm, methods involving USB had a higher diagnostic yield than those that did not (91% vs. 50%, respectively, *p* = 0.015). In terms of safety, patients who underwent USB developed no complications, even though 38.5% (10/26) of the lesions in the USB group were located within 10 mm of the pleura. Cryobiopsy has recently been used to obtain samples^12–14^, but this procedure carries bleeding and pneumothorax risk, especially PPLs close to the pleura^15–17^. However, our recommendation is not for obtaining smaller specimens when a larger sample is accessible, because small specimens cause challenges in pathological diagnosis and may result in problems related to tumor heterogeneity or sampling error. Some tumors in patients in the USB group were biopsied using USB and subsequently using standard-sized forceps, as described in a previous report^18^. However, our results showed particularly that for small distal PPLs, conventional methods could not capture malignant lesions in approximately half of the patients in the non-USB group in the first place. For some patients with small distal PPLs, only small samples were obtained. In the present study, although the difference was not significant, the “within” EBUS image was obtained only in 32% in the USB group, which seems to be lower than that in the non-USB group (56.5%). In addition, the diagnostic yield for lesions with “adjacent to” or “no signal” EBUS image in the USB group seems to be surprisingly high. We speculate that the smaller target size in the USB group might be the reason for the low number “within” EBUS findings. On the other hand, the USB method allowed the bronchoscope to reach closer to the target, and the DOM provided some information on the angle and distance toward the targets. These might help to direct the forceps toward the target for biopsy, even if the EBUS was adjacent to or no signal. Collectively, we propose that even with ultrathin bronchoscopy, suitable samples can be provided for NGS analysis, with superior accessibility, bronchial selectivity, and safety, for patients with small distal PPLs^6,7^.

The proposed SVA method enabled small specimens to meet the quantity criteria for the OTT test in this study. In clinical practice, samples are often sectioned at 5 μm thickness for cancer panel testing^19,20^. However, we recommend a 10 μm thickness not only for quantity but also for quality, as previously reported^21^. In addition, the problem of sequencing failure due to quantity is similar to that of the example of other than lung cancer^22,23^. Our proposed SVA method has the potential to improve the success ratio of sequencing tests of small, biopsied samples.

This study had two limitations. First, the diagnostic yield was retrospectively analyzed. Randomized prospective data are needed to eliminate selection bias. Second, although all samples met the quantity criteria, 4 of the 10 samples had less than 20% tumor cell content, which may be inadequate for NGS mutational analysis. However, because the lesions in these patients were located in the distal periphery, the conventional method alone could not capture tumor samples of these patients because of the low diagnostic rate and the risk of pneumothorax. It is important to have both options: a standard-sized forceps biopsy or cryobiopsy, even if the pathway is incorrect, or a small-sized forceps biopsy with a precise pathway.

## Conclusions

In conclusion, the pipeline of USB with SVA as a novel method that integrates an ultrathin bronchoscope, r-EBUS, and DOM for ultraselective navigation, and which uses more sections if the sample size is small, as well as amplicon-based NGS analysis, may be a better alternative for the molecular profiling in small PPLs.

## Supplementary Information

Supplementary Information 1.

Supplementary Video 1.

Supplementary Information 2.

## Data Availability

The datasets used and/or analyzed during the current study are available from So Takata (email: sotakata@imed3.med.osaka-u.ac.jp) upon reasonable request.
