# Supplementary material for: Proposal of a novel pipeline involving precise bronchoscopy of distal peripheral pulmonary lesions for genetic testing
Source: Sci Rep. 2022 Nov 17;12:19774. doi: 10.1038/s41598-022-24372-6 (PMC9672070; doi:10.1038/s41598-022-24372-6)
Supplement: Supplementary file 1 — Supplementary Information 1. [file 41598_2022_24372_MOESM1_ESM.pdf]

Supplementary Figure 1. Oncomine Target Test List

Hotspot genes, n=35				Copy number variants, n=19				Fusion drivers, n=23			
ALK	*BRAF*	*EGFR*	*ERBB2*	*ALK*	*BRAF*	*EGFR*	*ERBB2*	*ALK*	*BRAF*		*ERBB2*
FGFR2	*FGFR3*	*MET*	*PDGFRA*	*FGFR2*	*FGFR3*	*MET*	*PDGFRA*	*FGFR2*	*FGFR3*	*MET*	*PDGFRA*
AR	*CDK4*	*KIT*	*KRAS*	*AR*	*CDK4*	*KIT*	*KRAS*				
PIK3CA				*PIK3CA*							
RAF1	*RET*	*ROS1*						*RAF1*	*RET*	*ROS1*	
AKT1	*CTNNB1*	*DDR2*	*ERBB3*					*ABL1*	*AXL*	*ERG*	*ETV1*
ERBB4	*ESR1*	*GNAQ*	*GNA11*					*ETV4*	*ETV5*	*NTRK1*	*NTRK2*
HRAS	*IDH1*	*IDH2*	*JAK1*					*NTRK3*	*PPARG*		
JAK2	*JAK3*	*MAP2K1*	*MAP2K2*	*FGFR1*				*FGFR1*			
MTOR	*NRAS*	*SMO*									

The copy number variants descripted below
are reported as supplemental information.

CCND1 CDK6 FGFR4 MYC MYCN

Hotspot, CNV, Fusion, Hotspot + CNV, Hotspot + CNV + Fusion, Hotspot + Fusion, CNV+ Fusion

Genes identified by the oncomine target test.

Supplementary Figure 2. Representative image of a pathological specimen obtained using small forceps.

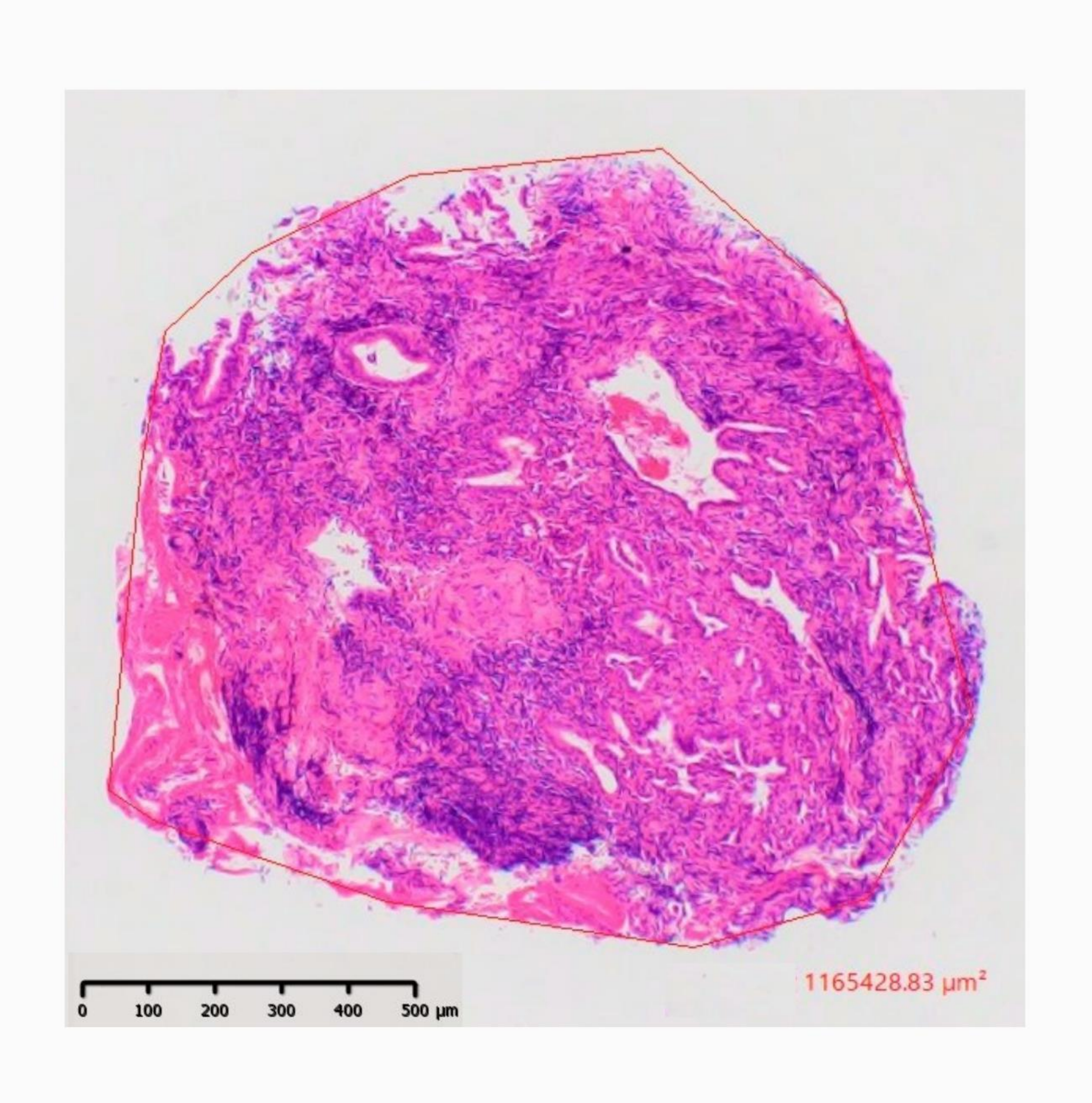

The area surrounded by the red outline (1.17 mm2 and H&E stain, ×400) was measured using CellSens Standard software (Olympus).

List of Supplementary Tables:

Supplementary Table 1. Thresholds of the Oncomine Target Test
Supplementary Table 2. Bronchoscopy related factors
Supplementary Table 3. Subgroup characteristics and bronchosopy related factors
Supplementary Table 4. Multivariate logistic regression analysis of diagnostic yield of subgroup
Supplementary Table 5. Histological diagnoses of samples in the USB group
Supplementary Table 6. The results of OTT and bronchoscopic evaluation in patients who underwent OTT

Supplementary Table 1. Thresholds of the Oncomine Target Test

**Tumor cell content**	**Concentration of nucleic acid (ng/μl)**		**Quality check of sequenced library**			**Call for aberration**			
	DNA	RNA	DNA library		RNA library	SNV/InDel		Fusion reads	CNV GAIN
			Length of read that met QV20	DNA%read	Total mapped reads	Coverage	Allele frequency		Lower confidence limit
≥ 20%	> 0.83	> 1.43	≥ 90 base pair	≥ 0.7	≥ 5000	≥ 347X	≥ 2.5–4.0%	≥ 40	≥ 4

Supplementary Table 2. Bronchoscopy related factors

	USB group (N = 25)	Non-USB group (N = 24)	P value
**Bronchoscopy related factors**			
Physicain_experience,years (median [IQR])	7.00 [6.00–8.00]	8.00 [6.00–8.00]	0.487
EBUS_image (%)			0.231
adjacent to	12 (48.0)	7 (30.4)	
no signal	5 (20.0)	3 (13.0)	
within	8 (32.0)	13 (56.5)	
Number of biopsy (median [IQR])	7.00 [6.00–8.00]	7.00 [5.25–9.00]	0.389
Procedure time minutes range (mean ± SD)	37.96 (13.72)	32.33 (10.43)	0.114
**The content of methods**			
Method (%)			
r-EBUS,ultrathin, and DOM (USB)	25 (100.0)	0 (0.0)	
r-EBUS + ultrathin	0 (0.0)	1 (4.2)	
r-EBUS + DOM	0 (0.0)	19 (79.2)	
Neither ultrathin and DOM	0 (0.0)	4 (16.7)	
Ultrathin scope use (%)	25 (100.0)	2 (8.3)	
Thin or Standard sized scope use (%)	13 (52.0)	22 (91.7)	
Bronchoscopic_navigation (%)			
VINCENT-BFsim	0 (0.0)	4 (16.7)	
VINCENT-DOM	10 (40.0)	10 (41.7)	
Ziostation2-DOM	15 (60.0)	10 (41.7)	
TBNA performed (%)	0 (0.0)	1 (4.5)	

DOM, direct oblique method; EBUS; endobronchial ultrasonography;
TBNA, transbronchial needle aspiration; USB, ultraselective biopsy.
Fisher's exact test or the chi-square test was performed for categorical variables as appropriate, and the Mann-Whitney U test was performed for continuous variables.

Supplementary Table 3. Subgroup characteristics and bronchosopy related factors

	USB group (N = 21)	Non-USB (N = 14)	P value
**Patient Characteristics**			
Age, years (mean ± SD)	69.95 (8.51)	69.43 (11.72)	0.879
Male (%)	11 (52.4)	9 (64.3)	0.727
Diameter of lesion, mm (mean ± SD)	16.86 (7.12)	18.64 (6.52)	0.458
Appearance on CT (%)			0.314
ground glass opacity	3 (14.3)	1 (7.1)	
part solid	2 (9.5)	4 (28.6)	
solid	16 (76.2)	9 (64.3)	
Bronchial generation (median [IQR])	9 (42.9)	8 (57.1)	0.629
Final diagnosis (%)	7.00 [6.00, 8.00]	6.50 [6.00, 8.00]	0.859
Adenocarcinoma			0.306
Large cell lung carcinoma	14 (66.7)	11 (78.6)	
Malignant lymphoma	2 (9.5)	0 (0.0)	
Metastatic lung carcinoma	0 (0.0)	1 (7.1)	
Non-small cell lung carcinoma,	0 (0.0)	1 (7.1)	
not otherwise specified	4 (19.0)	1 (7.1)	
Small cell lung carcinoma	1 (4.8)	0 (0.0)	
**Bronchoscopy related factors**			
Physicain_experience,years (median [IQR])	7.00 [6.00, 8.00]	7.50 [6.25, 8.00]	0.481
EBUS_image (%)			0.933
adjacent to	11 (52.4)	6 (46.2)	
no signal	4 (19.0)	3 (23.1)	
within	6 (28.6)	4 (30.8)	
Number of biopsy (median [IQR])	7.00 [6.00, 8.00]	7.50 [6.75, 9.00]	0.449
Procedure time minutes range (mean ± SD)	40.33 (13.16)	32.07 (9.08)	0.049
**The content of methods**			
Method (%)			
r-EBUS,ultrathin, and DOM (USB)	21 (100.0)	0 (0.0)	
r-EBUS + ultrathin	0 (0.0)	1 (7.1)	
r-EBUS + DOM	0 (0.0)	11 (78.6)	
Neither ultrathin and DOM	0 (0.0)	2 (14.3)	
Ultrathin scope use (%)	21 (100.0)	2 (14.3)	
Thin or Standard sized scope use (%)	12 (57.1)	12 (85.7)	
Bronchoscopic_navigation (%)			
VINCENT-BFsim	0 (0.0)	2 (14.3)	
VINCENT-DOM	7 (33.3)	5 (35.7)	
Ziostation2-DOM	14 (66.7)	7 (50.0)	
TBNA performed (%)	0 (0.0)	1 (8.3)	

DOM, direct oblique method; EBUS; endobronchial ultrasonography;
TBNA, transbronchial needle aspiration; USB, ultraselective biopsy.
Fisher’s exact test or the chi-square test was performed for categorical variables as appropriate, and the Mann-Whitney U test was performed for continuous variables.

Suplementary Table 4. Multivariate logistic regression analysis of diagnostic yield of subgroup

	Odds ratio (95% CI)	P-value
USB group	9.92 (1.68-94.98)	0.02
Procedure time	1 (0.91-1.08)	0.905

Supplementary Table 5. Histological diagnoses of samples in the USB group

	Histological diagnoses of samples in the USB group (n = 19)	
Only USB was performed	Small-sized forceps: positive	9
Standard-sized biopsy was added on USB	Small-sized forceps: positive, Standard-sized forceps: negative	3
	Small-sized forceps: negative, Standard-sized forceps: positive	1
	Specimens of both sizes were positive	1
	Samples were not separated	5
No histological diagnosis	Cytology finding: positive	3
	Cytology finding: negative	3

USB, ultraselective biopsy.

Supplementary Table 6. The results of OTT and bronchoscopic evaluation in patients who underwent OTT

No	DNA liquid volume （uL）	DNA concentration (ng/uL)	RNA liquid volume （uL）	RNA concentration (ng/uL)	DV200	Cumulative area	Areas of samples (mm^2) from H&E-stained samples used in practice	Total areas (mm^2) of H&E-stained samples used in practice	Area of each sample (mm^2) just before sectioning	Total area (mm^2) just before sectioning	Number of biopsied samples	Lesion diameter (mm^2)	Apearance on CT	Echo sign	Bronchus sign	Generation	Immuno histochemistry before sectioning
1	34	19.63	34	14.16	59	63.36	1.00, 0.96, 0.94, 0.96, 1.65, 0.48, 0.62, 0.27	6.88	0.54, 0.89, 0.56, 0.77, 0.25, 0.50, 0.43, 1.34	5.28	8	11	Solid	Adjacent to	-	9	Napsin A, p40, ALK, PD-L1
2	34	18.13	35	36.42	55	76.7	0.88, 1.27, 1.65, 2.35, 1.75	7.9	1.89, 1.83, 1.73, 1.54, 0.68	7.67	5	20	Solid	Within	+	7	CK5/6, p40, NapsinA, TTF-1, ALK, PD-L1
3	38	12.02	38	12.36	57	60.12	0.8, 1.4, 0.65, 0.76, 0.2, 0.02, 0.04	3.87	0.65, 0.63, 1.17, 2.42, 0.14	5.01	7	12	Solid	Adjacent to	-	6	TTF-1, ALK, PD-L1
4	34	10.93	34	9.5	59	58	0.82, 0.64, 0.89, 0.60, 1.6	4.55	0.94, 0.75, 1.78, 0.80, 0.56	5.8	5	11	GGO	Within	+	9	Napsin A, TTF-1, p40, ALK, PD-L1
5	37	4.45	38	5.06	46	24.4	1.90, 0.10, 0.42, 0.54	2.96	0.54, 0.36, 0.04, 1.5	2.44	4	19	Partly solid	No signal	-	10	EVG, ALK, CD31, D2-40, PD-L1
6	32	23.87	32	16.08	49	40.5	3.01, 0.95, 0.31, 0.44, 1.16, 1.07, 0.47	7.47	0.82, 0.03, 0.08, 0.09, 0.60	1.62	7	17	Solid	Adjacent to	-	6	D2-40, CD31, ALK, PD-L1
7	32	3.19	32	1.81	47	32.7	1.19, 1.26, 1.6, 0.40, 0.54, 0.36, 0.72, 0.59, 1.49	8.15	0.65, 0.14, 0.45, 0.64, 1.1, 0.29	3.27	9	8	Solid	Adjacent to	-	7	CK5/6, Napsin A, TTF-1, p40, ALK, PD-L1
8	36	10.75	34	7.99	52	54	0.50, 1.26, 1.33, 0.67, 0.73	4.5	0.50, 1.26, 1.33, 0.67, 0.73	4.5	5	10	Solid	Within	-	6	Napsin A, TTF-1, p40, ALK, EGFR covas, PD-L1
9	34	8.8	34	8.94	46	44.28	0.11, 0.91, 0.79, 1.10, 0.56, 0.23	3.69	0.11, 0.91, 0.79, 1.10, 0.56, 0.23	3.69	6	14	Solid	Adjacent to	+	7	CK5/6, Napsin A, TTF-1, p40, ALK, PD-L1
10	35	22.94	33	2.05	49	23.94	0.96, 0.21, 0.16, 0.54, 0.59, 0.42, 0.51	3.33	0.35, 0.45, 0.21, 0.32	1.33	7	30	Solid	Adjacent to	+	8	CK, CAM5.2, TTF-1, NapsinA, CK5/6, p40, synaptophysin, LCA, PD-L1
