# Supplementary material for: Proposal of a novel pipeline involving precise bronchoscopy of distal peripheral pulmonary lesions for genetic testing
Source: Sci Rep. 2022 Nov 17;12:19774. doi: 10.1038/s41598-022-24372-6 (PMC9672070; doi:10.1038/s41598-022-24372-6)
Supplement: Supplementary file 3 — Supplementary Information 2. [file 41598_2022_24372_MOESM3_ESM.docx]

Supplementary Video.

**The direct oblique method (DOM).** In this video, we demonstrated DOM procedure on Zionsation2. The bifurcations are evaluated using transverse and longitudinal sections of oblique computed tomography (CT) images.
